# Age-adjusted ELISA cutoffs for Johne’s disease in young adult dairy cattle using Bayesian nonparametric models

**DOI:** 10.3389/fvets.2026.1845826

**Published:** 2026-06-30

**Authors:** Mooyoung Jung, Gi-Won Park, Chan-Lan Kim, Han Gyu Lee, Tai-Young Hur

**Affiliations:** 1 Dairy Science Division, National Institute of Animal Science, Rural Development Administration, Cheonan, Republic of Korea; 2 College of Veterinary Medicine, Jeonbuk National University, Iksan, Republic of Korea; 3 Division of Animal Diseases & Health, National Institute of Animal Science, Rural Development Administration, Wanju, Republic of Korea

**Keywords:** dairy cows, ELISA, herd management, Johne’s disease, surveillance

## Abstract

Johne’s disease (JD), caused by *Mycobacterium avium* subsp. *paratuberculosis*, presents significant economic and health challenges for dairy farms globally. Enzyme-linked immunosorbent assay (ELISA) is commonly utilized for herd-level JD surveillance because of its cost-effectiveness and rapid execution, although its low sensitivity in young cattle complicates control efforts. This retrospective open-cohort study evaluated whether age-adjusted interpretation of longitudinal ELISA sample-to-positive (S/P) ratios during young adult age (24 to 48 months of age) could support risk stratification for subsequent JD seroconversion in a commercial dairy herd. A total of 500 dairy cows in a single herd with repeated serum ELISA testing between July 2018 and February 2026 were included. Age-adjusted cutoff patterns were estimated using linear, quadratic, and flexible spline Bayesian nonparametric models based on test-negative observations. Cows were classified according to longitudinal S/P ratio patterns during young adult age as below cutoff, non-consecutive exceedance, or consecutive exceedance. A landmark survival analysis from 48 months was performed to evaluate seroconversion risk. Overall, 43 of 500 cows (8.6%) became JD-seropositive. The performances across three models were comparable, with a high negative predictive value (96.5%) but low positive predictive value (about 11%). Using the linear model, cows with consecutive exceedances showed a higher hazard of seroconversion (hazard ratio 4.61, 95% CI: 1.22–17.40, *p* = 0.024) compared to the below cutoff group. These findings suggest that age-adjusted longitudinal S/P ratio patterns may be practical for subsequent JD seroconversion risk. However, the results are exploratory and require validation in diverse herds and against confirmed infection outcomes.

## 1 Introduction

Johne’s disease (JD), also known as paratuberculosis, is a chronic enteric infection in ruminants caused by *Mycobacterium avium* subspecies *paratuberculosis* (MAP), a highly resilient pathogen (1). In dairy cattle, JD incurs substantial economic losses, estimated at $33 per cow annually, primarily due to reduced milk yield, weight loss, premature culling, and increased mortality (2). JD remains widespread globally; a 2018 international survey reported herd-level prevalence estimates exceeding 40% in dairy herds in 13 of 27 surveyed countries, while in the Republic of Korea, herd-level prevalence has been reported at 1–10%, with within-herd prevalence ranging from 10 to 15% (3).

Controlling JD is challenging because of its slow, insidious onset and long incubation period—approximately 5 years in cattle and 2 years in small ruminants (4). The disease progresses through three clinical stages: latent non-shedding, light-shedding, and heavy-shedding, with the latter marked by substantial fecal MAP excretion and environmental contamination (5). Several diagnostic tools, including serum or milk enzyme-linked immunosorbent assay (ELISA), interferon-gamma assay, and fecal PCR, are available. However, the accuracy of these tests varies depending on the clinical stage of the disease (6).

The ELISA test is widely used for herd-level screening because of its cost-effectiveness, rapid turnaround, and suitability for large-scale testing (7, 8). Although ELISA is often used alongside others such as fecal culture and PCR, reliance on ELISA alone in young cows or in cows at the subclinical stage is limited because of its low sensitivity in early infection (6, 7, 9, 10). To overcome this limitation, some researchers recommend regular testing to improve the detection rate (11, 12). Furthermore, quantitatively interpreting ELISA results (using sample-to-positive [S/P] ratios) in combination with the qualitative interpretations (seropositive or seronegative) may provide additional information for monitoring subsequent ELISA seroconversion in cows (7).

A previous study suggests that the detectability of MAP infection in earlier stages increases with age based on milk ELISA and fecal culture, whereas detection of the infectious stage was not affected by cow age (13). Accordingly, we hypothesized that age-adjusted longitudinal S/P patterns during early adulthood might provide risk stratification of subsequent ELISA seroconversion. This study retrospectively analyzed routine serum ELISA results from a commercial dairy herd in the Republic of Korea. This brief research report aimed to evaluate whether age-adjusted longitudinal ELISA S/P ratio patterns during 24–48 months of age could stratify the risk of subsequent ELISA seroconversion in a single commercial dairy herd. The study was designed as an exploratory herd-level risk-stratification analysis, not as a validation of ELISA against confirmed MAP infection status. In this open-cohort study, repeated serum ELISA results from a single commercial herd were retrospectively analyzed to derive age-adjusted S/P cutoff patterns during 24–48 months of age and to evaluate their utility for subsequent ELISA-seroconversion risk stratification after 48 months.

## 2 Materials and methods

### 2.1 Study herd, surveillance program, and study design

This retrospective study was conducted in a commercial dairy herd located in Cheonan-si, Chungcheongnam-do, Republic of Korea. The herd conducted disease prevention programs two to four times annually with serum ELISA testing for brucellosis, foot-and-mouth disease, and JD. No positive findings for infectious diseases other than JD were detected during the study period.

This study was designed as a single-herd, open-cohort analysis of repeated JD ELISA results collected from July 2018 to February 2026. In this open-cohort setting, cows were not enrolled simultaneously; rather, they entered the cohort surveillance at the time of their first available JD ELISA test and exited because of culling, sale, death, or the end of observation, resulting in staggered entry and unequal follow-up durations. All cows born and raised on the farm, were participated in this repeated surveillance testing.

### 2.2 Serum ELISA procedures and study definitions

Blood samples were collected via jugular venipuncture into serum-separating tubes, centrifuged at 1,600 × g for 10 min, and analyzed using a commercial ELISA kit (*Mycobacterium paratuberculosis* Antibody Test Kit; IDEXX Laboratories Inc., Westbrook, ME, United States) according to the manufacturer’s instructions. ELISA results were expressed as sample-to-positive (S/P) ratios (%) and were retained as continuous variables for statistical analyses.

At each testing occasion, results with an S/P ratio ≥55% were considered test-positives, whereas all others were considered test-negatives. Individual cows were classified as JD-seropositive if they had at least one ELISA result ≥55% S/P ratio during the follow-up, whereas cows with exclusively < 55% S/P ratio results were classified as JD-seronegative. JD-seropositive cows were culled immediately after detection according to farm practice. Seroconversion was defined as the first occurrence of an ELISA-seropositive result during follow-up, with event timing assigned to the corresponding testing occasion.

### 2.3 Study population and analytic sets

A total of 6,381 ELISA test records from 599 cows (457 purebred Holstein and 142 purebred Jersey) were initially available. To focus on longitudinal analysis during young-adult the analytic dataset was restricted to cows first tested at ≤24 months of age.

Consequently, the main longitudinal dataset comprised 5,104 ELISA test records from 500 cows (368 Holsteins and 132 Jerseys). This dataset consisted of repeated test-level observations, including 4,783 test-negative results for 457 JD-seronegative cows. In addition, 43 seropositive cows contributed 321 test records, which included 278 test-negative results prior to seroconversion and 43 test-positive results at the time of seroconversion.

For survival analysis, a separate analytic dataset derived from the main dataset was constructed using a landmark design at 48 months of age. Only cows with at least one ELISA test at ≥48 months of age were included. In this dataset, each cow contributed a single animal-level observation for time-to-event analysis. The survival dataset comprised 190 cows, including 174 JD-seronegative (censored) and 16 JD-seropositive (event) animals. At the test-record level, this corresponded to 3,266 observations (3,250 test-negative and 16 test-positive results).

### 2.4 Analytical framework

The analysis proceeded in three stages. First, descriptive statistics were calculated at the animal level using the full longitudinal dataset (5,104 test records from 500 cows), including breed (Holstein or Jersey), age at initial test (months), and duration of testing (months).

Second, age-adjusted ELISA S/P ratio cutoff patterns were estimated using 5,061 test-negative observations, excluding the 43 test-positive results. All available longitudinal observations across ages were used to model age-dependent ELISA patterns, particularly within the 24 to <48 months of age window.

Third, for risk stratification, cows were classified based on their ELISA results between 24 and <48 months of age using age-adjusted cutoffs derived from the selected model. Three groups were defined: (i) below cutoff (no exceedance), (ii) non-consecutive exceedance (at least one exceedance without consecutive occurrences), and (iii) consecutive exceedance (two or more successive exceedances). For survival analysis, follow-up began at the 48-month landmark and continued until seroconversion or censoring at the last available test. This landmark design was applied to avoid immortal time bias.

### 2.5 Statistical analysis

Descriptive statistics were calculated at the animal level, summarizing cow counts, initial test ages, testing durations and total testing counts. Also, the proportions of Holstein and Jersey cows and the cows born in heat season (June to August, as generally defined in the Republic of Korea) and the cows born in non-heat season (not born from June to August). Continuous variables were represented as mean ± standard deviation, along with medians and interquartile ranges. Comparisons between JD-seropositive and JD-seronegative cows, as well as within JD-seropositive cows, were performed using the Wilcoxon rank-sum test, and proportional distributions were compared using the chi-square test.

Age-adjusted receiver operating curve analysis was conducted using Bayesian nonparametric regression implemented in the ROCnReg package (version 1.0–9) in R. The conditional distribution of S/P ratios among seronegative animals was modeled using a Dirichlet-process mixture of normal regressions (14), allowing flexible estimation of age-dependent marker distributions. Three alternative specifications for the age effect were evaluated to model robustness under different functional assumptions: (i) linear model: mean = β0 + β1(age at test), (ii) quadratic model: mean = β0 + β1(age at test) + β2(age at test^2^), (iii) flexible spline model: mean = β0 + ƒ(age at test).

Posterior inference was obtained via Markov chain Monte Carlo sampling using default ROCnReg settings. Convergence was assessed through visual inspection of trace plots and stability of posterior summaries. Age-adjusted optimal cutoffs were determined by maximizing Youden’s index (sensitivity + specificity − 1). Model performance was assessed by sensitivity, specificity, predictive values, and area under the curve with 95% confidence intervals estimated using the DeLong method (15).

Kaplan–Meier survival analysis (16), using the survival package (version 3.8–3) in R, was performed to evaluate risk stratification based on longitudinal ELISA results using a landmark design. When applying the age-adjusted cutoff models, the performances of linear, quadratic, and flexible spline models were considered. When model performance was comparable across the three models, the linear model was selected for its simplicity and interpretability. The classification window was defined as 24 to <48 months of age. According to age-adjusted cutoffs derived from the prior Bayesian nonparametric model, cows were classified into three groups: (i) below cutoff (no exceedance), (ii) non-consecutive exceedance (at least one exceedance without consecutive occurrences), and (iii) consecutive exceedance (two or more successive exceedances). Follow-up began at 48 months of age and continued until seroconversion or the last available test. Group differences were assessed using the log-rank test, and hazard ratios were estimated using Cox proportional hazards models (17). The proportional hazards assumption was evaluated using Schoenfeld residuals.

All analyses were performed in R (version 4.3.3; R Foundation for Statistical Computing, Vienna, Austria), and statistical significance was set at *p*-value < 0.05.

## 3 Results

In the first stage, descriptive statistics were summarized at the animal level based on the full longitudinal dataset comprising 5,104 test records from 500 cows. Overall, the open-cohort study for descriptive statistics at the animal level, 43 (8.6%) were confirmed as JD-seropositive (Table 1). The proportion of Holstein and Jersey breeds was comparable within the JD-seropositive and JD-seronegative groups, as were the proportion of cows born in heat and non-heat seasons. Although JD-seropositive cows underwent fewer tests than JD-seronegative cows, the age at initial test and the duration of testing were similar between the two groups. The mean age at seroconversion of JD-seropositive cows was 37.3 months (95% confidence interval, 28.6 to 46.0 months) (data not shown). When stratified by breed, the mean age at seroconversion was 34.7 months (95% confidence interval, 25.9 to 43.5 months) in 32 seropositive Holstein cows and 52.1 months (95% confidence interval, 28.8 to 75.4 months) in 11 seropositive Jersey cows (Wilcoxon rank-sum test; *p* = 0.092).

**Table 1:** Summary of serum ELISA test results and Johne’s disease prevalence in the dairy herd during the test period.

Variables	JD-seronegative	JD-seropositive	*p*-value
Number of cows (cows)	457	43	
Number of Holstein cows [Cows (%)]	336 (91.3%)	32 (8.7%)	1.000 ^a^
Number of Jersey cows [Cows (%)]	121 (91.7%)	11 (8.3%)	
Number of cows born in heat season [Cows (%)]	116 (89.9%)	13 (10.1%)	0.471 ^a^
Number of cows born in non-heat season [Cows (%)]	341 (91.9%)	30 (8.1%)	
Total testing counts (times)	10.46 ± 6.84 [10.00, 12.00]	7.47 ± 5.15 [7.00, 8.00]	0.007 ^b^
Age at the initial test (months)	8.01 ± 4.13 [7.00, 3.00]	9.74 ± 5.62 [8.00, 3.50]	0.080 ^b^
Duration of testing (months)	32.09 ± 24.25 [31.00, 37.00]	29.40 ± 25.17 [21.00, 40.00]	0.423 ^b^

The numbers of Holstein cows and Jersey cows as well as the cows born in heat season (born in June to August) and non-heat season (not born in June to August) are expressed as the number of cows (percentages which were calculated in the same rows). The total testing counts, age at the initial test, and the duration of testing are expressed as mean ± standard deviation [median, interquartile range].

a, chi-square test; b, Wilcoxon rank-sum.

JD, Johne’s disease.

**Figure 1:**
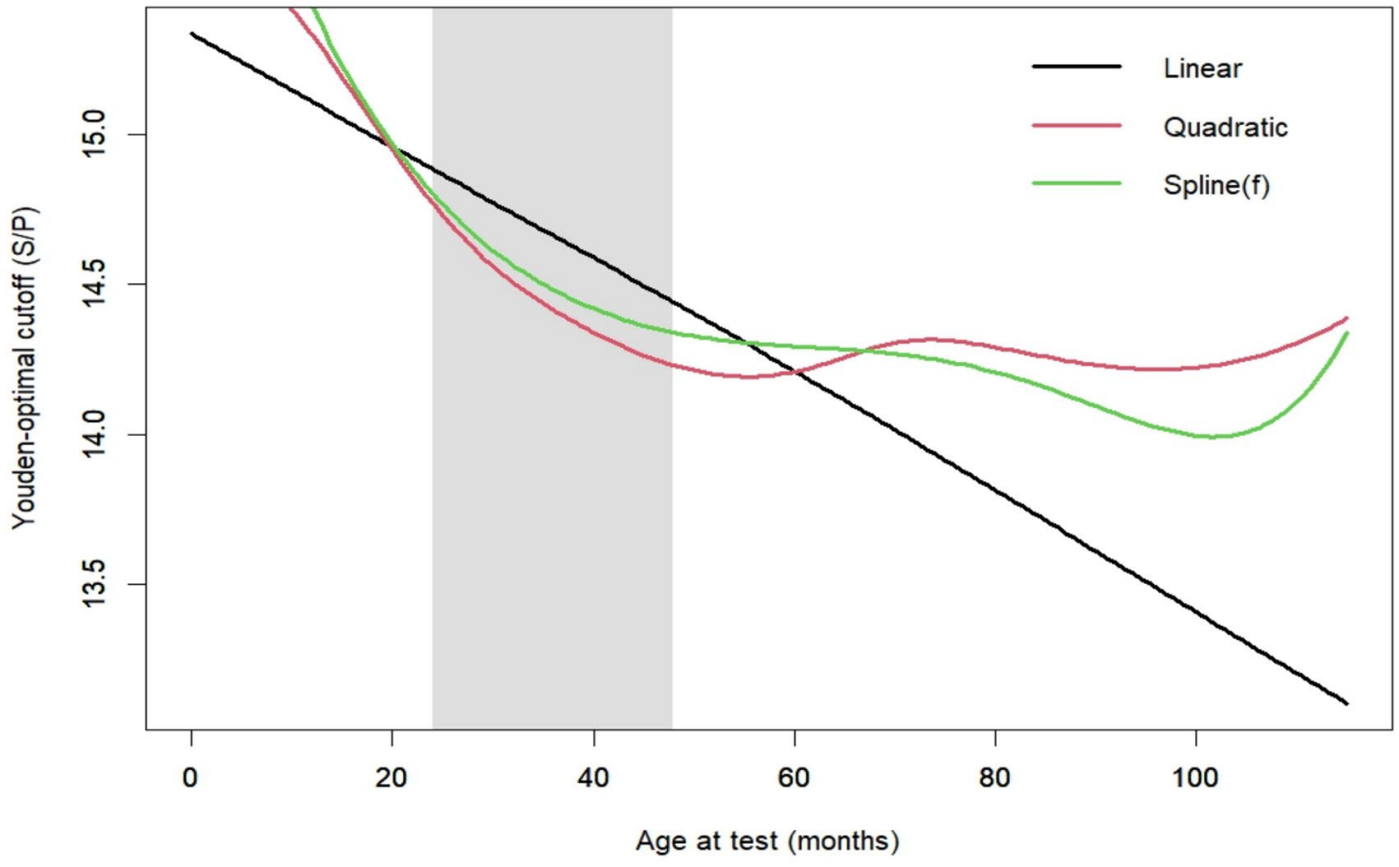
Age-adjusted cutoffs at the age of test calculated by the Bayesian bootstrap model using the Youden Index. Black, red, and green lines demonstrate the age-adjusted cutoffs using linear, quadratic, and flexible spline models, respectively. The highlighted age range is 24–48 months, representing the young adult ages focused on in this study.

In the second stage, age-adjusted ELISA S/P ratio cutoff patterns were estimated using 5,061 test-negative observations after excluding the 43 test-positive results, Across the 24 to <48 months of age window, the performances of linear, quadratic, and flexible spline models were comparable (Table 2). The positive predictive values of linear, quadratic, and flexible spline models were similarly 11.3, 11.1, and 11.5%, respectively, with identical negative predictive values of 96.5%. The resulting age-adjusted cutoff curves (S/P ratio, %) for these three models are shown in Figure 1, with specific young adult age ranges highlighted.

**Table 2:** Summary of model performance using age-adjusted cutoffs for 24–48 months of age.

Models	AUC (95% CI)	Sensitivity	Specificity	Accuracy	Mean error	PPV	NPV
Linear	0.615 (0.547–0.685)	0.437	0.817	0.798	0.202	0.113	0.965
Quadratic	0.615 (0.547–0.685)	0.448	0.805	0.787	0.213	0.109	0.965
Flexible spline	0.615 (0.547–0.685)	0.437	0.814	0.794	0.205	0.111	0.965

AUC, area under curve; CI, confidence interval; PPV, positive prediction value; NPV, negative prediction value.

In the third stage, risk stratification was performed using age-adjusted cutoffs derived from the selected model. The linear age-adjusted cutoff model was chosen due to its comparable performance to the quadratic and flexible spline models and its greater simplicity. Based on this model, animals were classified into three groups according to longitudinal S/P ratio patterns, with the following distributions by breed: below cutoff (Holstein: 59; Jersey: 18), non-consecutive exceedance (Holstein: 67; Jersey: 3), and consecutive exceedance (Holstein: 38; Jersey: 5). The distribution of animals across these risk groups differed significantly between breeds (chi-square test, *p* = 0.003). However, the proportion of animals that underwent seroconversion did not differ significantly between breeds (Holstein: 13/164; Jersey: 3/26; chi-square test, *p* = 0.540).

**Figure 2:**
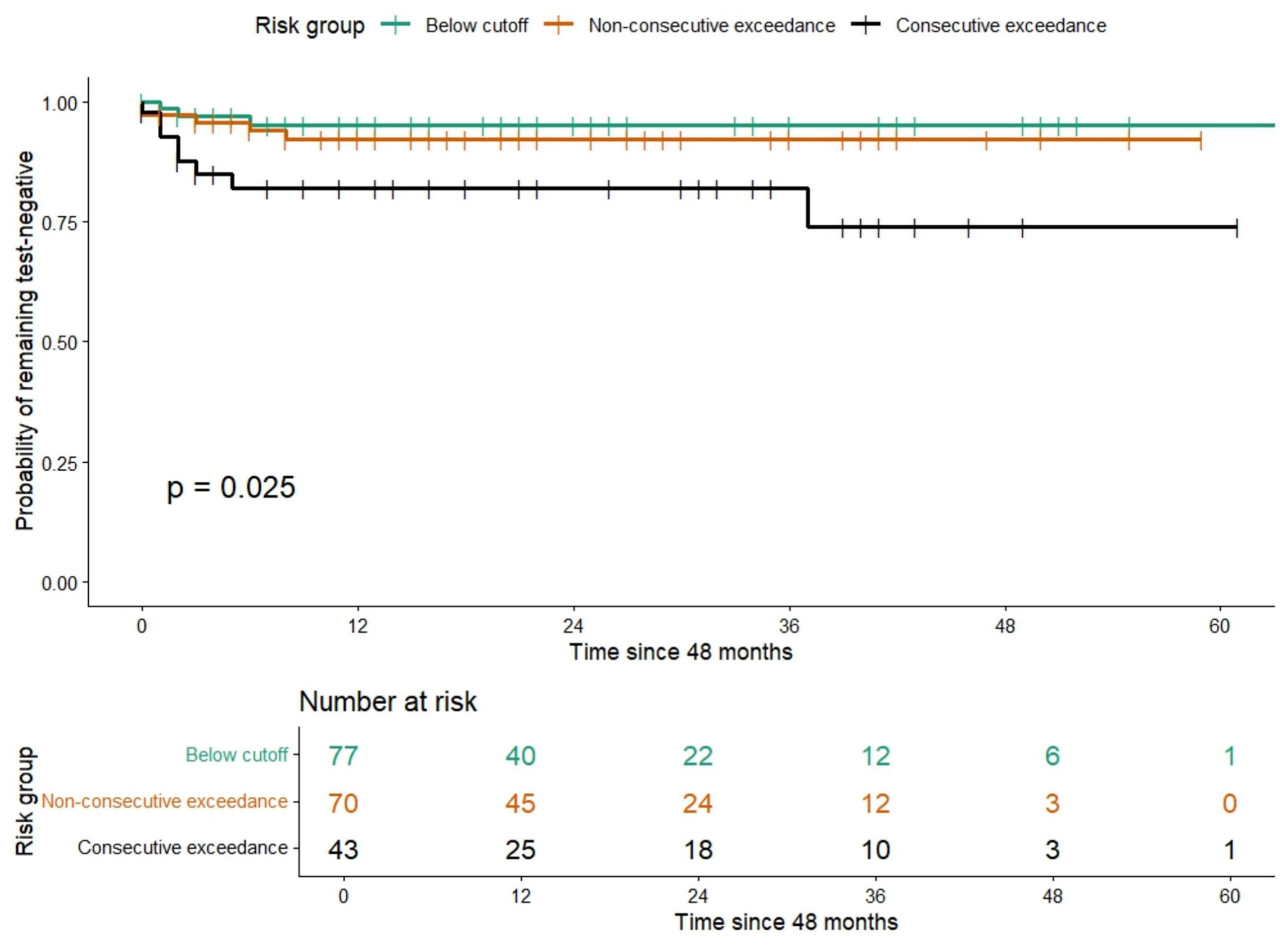
Kaplan–Meier Survival analysis for risk stratification using the linear Bayesian model. Green, orange, and black lines represent JD-seronegative survival for the below-cutoff, non-consecutive exceedance, and consecutive exceedance groups, respectively. The *p*-value displayed in the plot was calculated using the log-rank test. The numbers in the risk table represent the number of cows remaining test-negative for Johne’s disease in each group at each time point. The x-axis represents time since 48 months of age. JD, Johne’s disease.

For survival analysis, follow-up began at the 48-month landmark and continued until seroconversion or censoring at the last available test, in accordance with the predefined landmark design to avoid immortal time bias. Kaplan–Meier analysis showed a trend toward differences in JD-seronegative survival among the three groups defined by longitudinal S/P ratio patterns. The overall log-rank test reached conventional statistical significance (Figure 2; *p* = 0.025), whereas pairwise log-rank comparisons were not significant after adjustment for multiple testing. In the Cox proportional hazards model, animals with consecutive exceedance of the age-adjusted cutoff had a significantly higher risk of subsequent seroconversion compared to those consistently below the cutoff (hazard ratio = 4.61, 95% confidence interval: 1.22–17.40, *p* = 0.024). In contrast, animals with non-consecutive exceedance did not show a significant increase in risk (hazard ratio = 1.63, 95% confidence interval: 0.39–6.84, *p* = 0.501).

## 4 Discussion

This study examined longitudinal ELISA S/P ratio patterns in dairy cattle and explored whether risk stratification based on age-adjusted cutoff patterns may be associated with subsequent JD seroconversion. The findings suggest that age-adjusted interpretation of ELISA results has the potential to support risk stratification in routine surveillance, although the observed differences should be interpreted with caution. In particular, persistent elevation of ELISA responses, as measured using a commercial kit, may reflect progression toward detectable JD infection, whereas transient fluctuations appeared to have limited prognostic value.

The herd-level JD seropositivity rate (8.6%) was slightly lower than the reported 10–15% within-herd prevalence in the Republic of Korea (3), potentially due to effective JD management programs in this herd. The age of seroconversion aligns with known age ranges between 2.5 and 4.5 years (12). Although the Jersey cows have been reported to have a higher risk of JD seropositivity (18), breed predisposition was not evident in our study aligned with previous findings (19). This may suggest that breed-related differences in ELISA response patterns do not necessarily translate into differences in progression to seropositivity, although further investigation is warranted. The fewer tests in JD-seropositive cows compared to JD-seronegative cows may result from culling practices based on serum ELISA test results, which is an officially recognized alternative diagnostic method for fecal culture or PCR in Korea (20).

Because of slow-progression of JD, age is an important factor influencing detectability, with most cases identified in later stages of infection (11). Given the low detection rate in younger cows and its dependence on herd prevalence (21), this study focused on the young adult stage. In addition, immunological assays such as ELISA reflect host immune reactions that evolve over time, which limits their sensitivity in early or subclinical infection stages (13, 22). A central aspect of the present study is the use of age-adjusted ELISA S/P ratio cutoffs. Age was specifically considered because host immune responses to *Mycobacterium avium* subsp. *paratuberculosis* are known to vary over time, particularly during the transition from subclinical to detectable stages of infection. Recent studies have emphasized the importance of adjusting ELISA results for non-disease-related factors, such as milk yield and milk quality, to improve interpretation (23, 24). While those approaches focus on production-related variables, the present study aimed to account for age-related variation using longitudinal data. In this context, age-adjusted trajectories may provide a biologically relevant framework for interpreting serial ELISA results, although further comparative evaluation with other adjustment approaches would be needed. Accordingly, the age-adjusted cutoffs used in this study were developed to reflect age-related variation in ELISA responses across longitudinal observations, rather than relying on a single static threshold. This approaches were intended to better distinguish relatively low-risk animals during earlier stages of infection under field conditions.

While herd-level risk factors have been examined in several studies, cow-level risk stratification remains largely unexplored (25–27). The sequential serum ELISA testing is recognized as a practical management approaches in commercial herds (12), primarily identifying seropositive cows. Risk stratification approaches combining ELISA with fecal PCR has also been suggested (28), and Bayesian methods using ELISA results have been applied to estimate herd-level prevalence with high discriminatory performance (29). Compared to these approaches, the present study focuses on age-dependent longitudinal ELISA patterns at the individual animal level, which may be more applicable in herds where routine ELISA testing is conducted without confirmatory diagnostics. The relatively low positive predictive value observed in this study reflects the low prevalence of seroconversion, whereas the high negative predictive value (96.5%) suggests potential utility as a rule-out tool for identifying low-risk animals. However, these performance metrics should be interpreted within the context of herd prevalence and study design.

Although all three Bayesian models demonstrated comparable predictive performance, the linear model was selected for survival analysis due to its interpretability and ease of implementation in field settings. Given the minimal differences in model performance, the use of a simpler functional form was considered appropriate. The survival analysis corresponds to a landmark design, in which risk classification is determined based on ELISA results accumulated during the 24–48 month window, and JD-seronegative survival is subsequently evaluated from 48 months of age onward. This design ensures temporal separation between risk assessment and outcome evaluation, thereby minimizing time-dependent bias. Although the overall Kaplan–Meier comparison reached statistical significance, pairwise comparisons were not significant after adjustment. Although the Cox proportional hazards model identified a higher risk for cows with consecutive exceedances, the hazard ratio estimates were associated with wide confidence intervals. These findings likely reflect the limited number of events and should be interpreted cautiously. Nevertheless, the consistent elevation of the S/P ratio, rather than transient fluctuations, may be more relevant for identifying animals at increased risk of subsequent seroconversion. This interpretation is broadly consistent with previous work indicating that changes in S/P ratios between sequential ELISA tests among JD-seronegative cows have limited value for determining infection status (30), although longitudinal patterns may provide additional context for risk stratification. The magnitude of this association should be interpreted cautiously given the limited number of events.

Several limitations must be acknowledged. While highly specific, serum ELISA has relatively low sensitivity for early MAP infection (6), potentially leading to misclassification. False-positive reactions have also been documented in fecal culture-negative cows (30). Seroconversion in this study was defined as a field-based endpoint rather than confirmed infection status. Integrating cell-mediated diagnostics or molecular assays may improve classification accuracy in future studies. Furthermore, this study was conducted in a single herd, and validation across diverse management environments is necessary to generalize these findings. In addition, milk yield was not included in the present analysis due to the lack of test-day matched production data. Given that milk yield varies dynamically across lactation stages, the use of non-synchronous measurements could introduce bias. Future studies incorporating test-day milk yield data alongside ELISA measurements would allow a more precise evaluation of production-related effects on S/*p* values.

In conclusion, the findings of this study suggest that age-adjusted interpretation of longitudinal ELISA S/P ratios may be useful for risk stratification in JD surveillance. In particular, persistent exceedance of age-adjusted thresholds may be associated with increased risk of subsequent seroconversion. However, these findings should be considered exploratory and further validation in larger and more diverse populations is recommended before broader application.

## Data Availability

The raw data supporting the conclusions of this article will be made available by the authors upon reasonable request, subject to institutional legal and procedural guidelines.
